# Glycolysis and gluconeogenesis: A teaching view

**DOI:** 10.1016/j.jbc.2020.100016

**Published:** 2020-12-08

**Authors:** Félix Hernández

**Affiliations:** Department of Molecular Neuropathology, Centro de Biología Molecular Severo Ochoa, CBMSO, CSIC-UAM, Madrid, Spain

I read with interest the recent review “Tracking the carbons supplying gluconeogenesis” by Ankit M. Shah and Fredric E. Wondisford (1). The figures are clear, and they are a good teaching source. Nevertheless, I note some potential teaching issues as well as offer additional suggestions. It is well known and it is explained to our students that there are three reactions of glycolysis that are essentially irreversible: hexokinase, phosphofrutokinase-1, and pyruvate kinase. Thus, I would like to observe in relation to Figure 1 that

(1) With the first two reactions as from glucose to glucose-6-phosphate and from fructose-6-phosphate to fructose-1,6-bisphosphate, the enzymes in both directions should be labeled;
(2) With the reaction from phosphoenolpyruvate to pyruvate, there should be one single arrow;
(3) There should be another single arrow between pyruvate and oxaloacetate.

## Conflict of interest

The authors declare that they have no conflicts of interest with the contents of this article.
